# Correlates of residents' enrolment intention toward inclusive commercial health insurance in China: involvement, perceived benefit, perceived sacrifice, and government participation

**DOI:** 10.3389/fpubh.2023.1121783

**Published:** 2023-10-30

**Authors:** Tianyi Shen, Yinuo Wang, Jinping Xie, Xu Han, Rong Shao, Rong Jiang

**Affiliations:** ^1^Mudi Meng Honours College, China Pharmaceutical University, Nanjing, Jiangsu, China; ^2^School of International Pharmaceutical Business, China Pharmaceutical University, Nanjing, Jiangsu, China; ^3^The Research Centre of National Drug Policy and Ecosystem, China Pharmaceutical University, Nanjing, Jiangsu, China; ^4^Institute of Regulatory Science, China Pharmaceutical University, Nanjing, Jiangsu, China

**Keywords:** inclusive commercial health insurance, China, enrolment intention, involvement, perceived benefit, perceived sacrifice, government participation

## Abstract

**Background:**

As an application of inclusive finance in health insurance, inclusive commercial health insurance (ICHI) is a new public-private partnership-based health insurance scheme and has been vigorously promoted by the Chinese government in recent years to develop China Multi-level Health Insurance System, a system that aims to seek a mix of public and private sources to provide more affordable financial protection to all levels of society in line with their needs. However, the overall enrolment of ICHI scheme is still at a low level, and little is known about what influences residents' enrolment intentions. The aim of this study was to examine the multidimensional factors influencing residents' behavioral intentions and to develop a multivariate conceptual model to explore the psychographic process in the formation of enrolment intention.

**Methods:**

The empirical data used for model validation were obtained from a cross-sectional study conducted in Nanjing, China, a representative pilot city of ICHI scheme in 2022. Exploratory factor analysis, ANOVA, standard multiple regression, and hierarchical multiple regression were mainly employed for hypothesis testing.

**Results:**

The findings revealed that involvement, perceived benefit, and perceived sacrifice are all crucial psychographic process factors in the formation of residents' enrolment intentions. Government participation positively moderates the influence path of “perceived benefit—enrolment intention” but negatively moderates the path of “perceived sacrifice—enrolment intention”. Moreover, it was discovered that perceived benefit mediates the effect of involvement on enrolment intention, while perceived sacrifice does not.

**Conclusions:**

Improving residents' perceived benefit and involvement degree of the product, as well as reducing their perceived sacrifice, are both key to increasing their enrolment intentions. This study also points out that one of the main dilemmas in the current development of ICHI scheme is the low level of involvement among residents, and that optimizing the product design to make it more relevant to residents' lives is a more beneficial strategy to increase overall involvement.

## Background

Inclusive Commercial Health Insurance (ICHI) is a new type of public-private partnership-based voluntary health insurance scheme vigorously promoted by the Chinese government in recent years. It is also the application of “Inclusive Finance” in the field of health insurance. Inclusive finance is a core concept of finance that refers to equitable access for all levels of society, at affordable costs, to a wide range of financial services, provided by a variety of sound and sustainable institutions (1–3). The idea of inclusive finance came from the International Year of Microcredit (2005), launched by the United Nations and the World Bank to recognize the critical role inclusive finance plays in expanding access to financial services for low- and middle-income households as well as helping them in resisting risks (1).

ICHI scheme is city-customized, and its inclusiveness is mainly reflected in three aspects: (1) Low premium and community rating. ICHI scheme adopts community rating, and the average premium nationwide is <0.28% of disposable income per capita (2021). This makes ICHI affordable for almost all households, even those in poverty. (2) Low threshold for enrolment. There is no restriction on age or occupation and no strict restriction on pre-existing medical conditions, which makes ICHI accessible to all groups in society, especially to older adult groups and those with pre-existing health problems. (3) High benefit ceiling. As a complementary insurance to basic social health insurance, ICHI focuses on covering high out-of-pocket expenses (especially for serious and catastrophic diseases) after reimbursement by basic social health insurance, with a benefit ceiling of RMB 1 million. This will help most households to withstand the financial shock of catastrophic health expenditure due to serious diseases.

Public-private partnerships are the foundation on which ICHI scheme operates. Specifically, the underwriter of ICHI scheme is usually a co-insurer formed by several large insurance companies under the guidance of the local government, so, in essence, ICHI scheme is a private health insurance. The government makes overall planning or assists in formulating insurance guarantee plans, participates in product design and promotion, and provide supports such as sharing health insurance data. With the joint efforts of the government and insurance companies, as of 31 December 2021, ICHI scheme has been launched in 244 prefecture-level cities, with a cumulative total of about 140 million enrolments (including second-year renewals) and a total premium size of about RMB 14 billion (4).

From the perspective of insurance economics, the operation of such a low-premium insurance requires a large risk pool. However, the overall resident enrolment rate of ICHI scheme is only around 5% nationwide, and the enrolment rate in most cities has remained relatively low, which is not sufficient to form a sustainable premium pool to share the risk (4–6). In other words, higher resident enrolment will be key to the financially sustainable development of ICHI scheme. From the perspective of developing the broader health system, a higher enrolment rate will make ICHI scheme a core component of China Multi-level Health Insurance System, a system that seeks a mix of public and private sources to provides more supplementary insurance schemes on top of basic social health insurance, as ICHI scheme is expected to fill the gap between the “basic” protection of social health insurance and the “high-premium” protection of private health insurance. Furthermore, from the perspective of international impact, the sustainability of the ICHI scheme will provide a practical model for other social health insurance-led countries to link social insurance and private insurance, with the aim of alleviating the growing financial pressure of social health insurance as a single payer. Based on the above, it is of particular importance to further increase resident enrolment at this stage.

Since ICHI is a new type of insurance scheme originated in China, there is an absence of research specific to ICHI scheme in international context yet. Chinese scholars' literature on how to increase resident enrolment of ICHI scheme mainly focuses on theoretical research and macro data analysis. Most scholars focused on its special “dual supplier” and studied from the perspective of the government and insurance companies, mainly by analyzing the government's macro development data for products in representative cities (7, 8) and the operational effectiveness of inclusive public-private partnership model (9–11), as well as discussing the design logic of existing products and the operating model of insurance companies (5, 6, 12). Despite the optimization of public-private partnership model and insurers' operating models, as well as greater government advocacy to encourage residents into the ICHI system, many residents still choose to drop or refuse to enroll in ICHI scheme. This environment surrounding the ICHI system suggests the need to explore a new question. In the case of voluntary health insurance, the focus is not only on the sound operation of the supply side, but we need to be aware that residents' subjective behavioral intention to purchase is the direct internal motivation for their choice to enroll. Therefore, understanding the motivational factors and barriers that can explain residents' behavioral intentions toward ICHI from a demand-side perspective is essential to finding ways to increase the enrolment. In this context, this study aims to contribute to knowledge on this topic by identifying the multidimensional factors that influence residents' behavioral intentions toward ICHI scheme, exploring the multivariate psychographic process in the formation of their enrolment intention, and developing a conceptual model to describe the interrelationships between the key psychographic factors. Finally, feasible suggestions for health policymakers and promoters to increase ICHI enrolment among residents would be put forward according to the research results.

## Research factors and theoretical framework

Given that ICHI has stronger private health insurance attributes from a demand-side perspective, the theoretical part of this study was based on broader research on private health insurance to explore the factors influencing residents' enrolment intention. Particularly, the following variables are examined and explained in the following paragraphs: enrolment intention, perceived value variables (i.e., perceived benefit and perceived sacrifice), involvement and government participation.

Enrolment intention is the dependent variable in this study which refers to the subjective tendency of residents to invest in ICHI scheme, and it also directly reflects the possibility of making the enrolment decision (13, 14). Previous literature has suggested that people would have a greater intention to enroll in health insurance if they perceive the potential benefits outweigh the costs (15). This trade-off of benefit and sacrifice often relies on Perceived Value Theory in marketing. According to Zeithaml (16), perceived value is defined as the overall evaluation of the product by consumers after comprehensively considering the perceived benefits and the sacrifices, and it can influence consumers' purchase behaviors. In the field of studies of health insurance, perceived value as a pre-influencing factor of behavioral intention has been used in several studies and a causal relationship was found between them (15, 17, 18).

In practical empirical studies, the perceived value variable can be divided into perceived benefit and perceived sacrifice in order to better measure it (16, 19). Many studies based on the above classification of perceived value has suggested that perceived benefit has a positive influence on behavioral intention to invest, while perceived sacrifice has a negative influence (20–22). Thus, this study proposed the following hypotheses:

**H1a:** Perceived benefit positively influences residents' enrolment intention toward ICHI scheme.
**H1b:** Perceived sacrifice negatively influences residents' enrolment intention toward ICHI scheme.

However, the use of perceived value as an independent variable to measure behavioral intention assumed perfect and equal knowledge amongst individuals who can make comprehensive consideration and trade-off between benefits and costs. In fact, most consumers are often in a state of information asymmetry and do not have a strong subjective intention to collect and process more information regarding products that are not of interest to them or that they feel less relevant to. Therefore, it is difficult for them to make such a comprehensive consideration. To solve this problem, an antecedent factor of perceived value is needed to describe consumers' perceived relevance to the product and to explain the process of information collection and processing. Based on literature reviews, Involvement was considered a suitable antecedent factor which is defined as a motivational variable reflecting the degree of a person's perceived relevance of the object to the individual based on his inherent needs, values, and interests (23). It has been proposed by precious literature that, when making decisions, individuals with deeper involvement tend to search for more information and more proactively (24), process relevant information more systematically (25), and will have a more comprehensive consideration on the strengths and weaknesses of possible alternatives (26). Therefore, the degree of a consumer's involvement will have an impact on information processing and consideration of benefits and sacrifices (26–28). Moreover, several studies have demonstrated that involvement has a positive impact on consumers' perception of benefits, while their perception of sacrifice decreases with increasing involvement and duration (29, 30). Therefore, the following hypotheses were proposed:

**H2a:** Involvement positively influences residents' perceived benefit of enrolling in ICHI.
**H2b:** Involvement negatively influences residents' perceived sacrifice of enrolling in ICHI.

In addition to the impact of involvement on perceived value, involvement was often seen as an important psychographic construct due to its potential influence on consumer's behavior with respect to decision making and purchase intention (31, 32). It has been suggested in many empirical studies that involvement has a positive influence on consumers' behavioral intention to purchase (33–35). Therefore, we proposed the following hypothesis:

**H3:** Involvement positively influences residents' enrolment intention toward ICHI scheme.

Furthermore, according to Liang and Lai (36), it was believed that consumers will pass “Problem recognition—Information collection—Evaluation of alternatives—Purchase process evaluation—Post-purchase services evaluation” five stages, and finally form behavioral intentions and make a purchase decision. In this study, involvement refers to residents' perception of the relevance to ICHI based on their inherent needs, values, and interests, including the process of problem recognition and information collection. In contrast, perceived value refers to a comprehensive evaluation of ICHI scheme after weighing the benefits and sacrifices. This evaluation includes alternatives before purchase, the purchase process, and post-purchase services, the last of which is especially vital for insurance products. Therefore, we deduced there was an influence pathway of “involvement—perceived value—enrolment intention” between the three variables, and similarly, many studies have shown that perceived value mediates the effect of involvement on behavioral intention (37–39). Therefore, the following hypotheses were proposed:

**H4a:** Perceived benefit mediates the effect of involvement on enrolment intention toward ICHI scheme.
**H4b:** Perceived sacrifice mediates the effect of involvement on enrolment intention toward ICHI scheme.

In addition, according to previous research conducted for this study, it has been clarified that ICHI scheme is subject to a certain degree of government intervention. Many studies have shown the role of government as promoter and regulator of ICHI scheme in enhancing residents' perception of benefits, reducing their perceived risk, and thus increasing their enrolment intention (4, 9, 11). Thus, this study proposed the following hypotheses:

**H5a:** Government participation moderates the relationship between perceived benefit and enrolment intention toward ICHI scheme.
**H5b:** Government participation moderates the relationship between perceived sacrifice and enrolment intention toward ICHI scheme.

**Figure 1:**
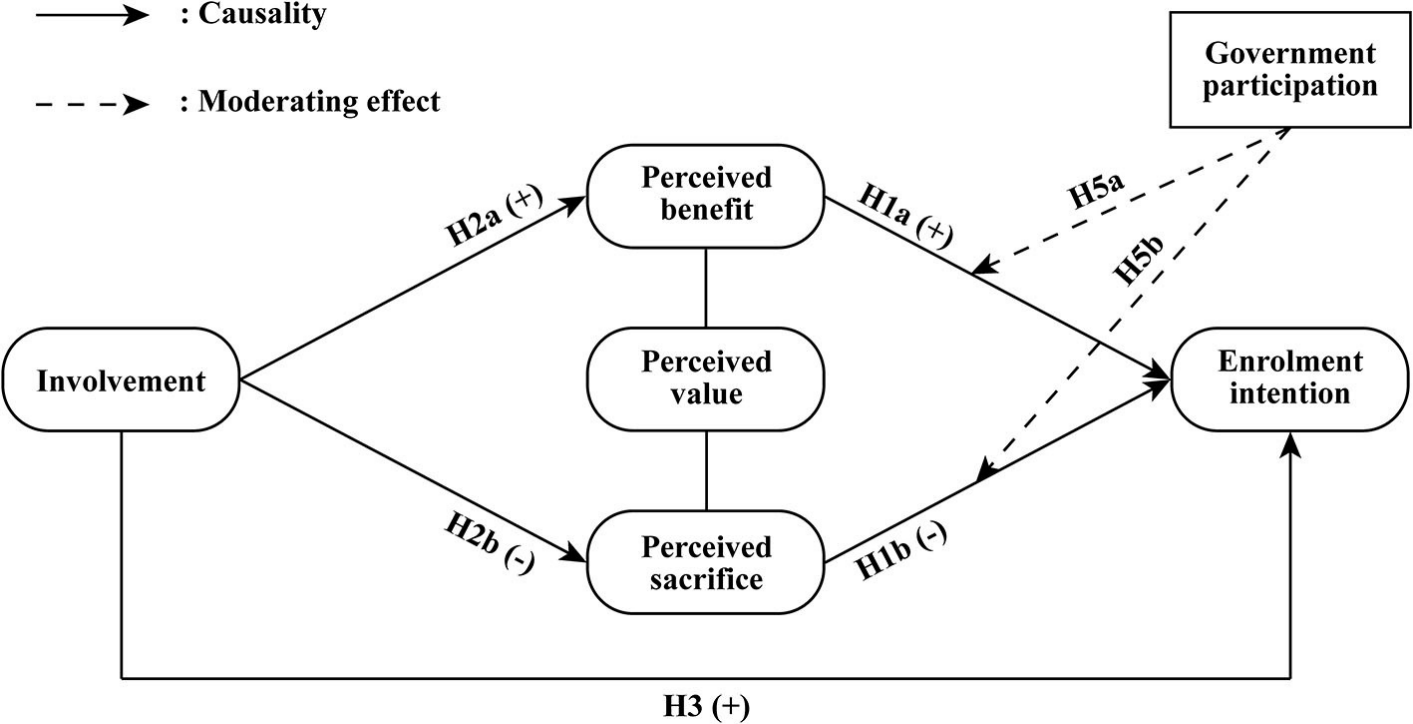
Hypothesized model to be tested.

Figure 1 shows the theoretical model proposed in this study.

## Methods

### Measure

An offline questionnaire was developed to measure the variables and test the hypotheses. The questionnaire consists of two parts involving demographic questions and the measurement of model construct variables. All survey items in the latter part utilized a 5-point Likert scale ranging from “agree strongly” to “disagree strongly”. They were developed based on existing literature, including five constructs (see Table 1):

**Table 1:** Factor loadings, mean, and standard deviation (SD) of each survey item's mean.

**Constructs**	**Measurement dimensions**	**Codes**	**Factor loadings**				**Mean**	**SD**
			**F1**	**F2**	**F3**	**F4**		
Involvement α = 0.937 KMO = 0.903^***^ Variance explained = 67.80 (%)	Advertising involvement	A1	0.874				2.99	1.046
		A2	0.910				3.00	1.021
		A3	0.886				3.02	1.043
	Product involvement	A4		0.925			2.98	1.027
		A5		0.881			3.05	1.002
		A6		0.835			2.83	1.059
	Enrolment-decision involvement	A7			0.713		3.05	0.913
		A8			0.608		3.19	0.876
Perceived benefit α = 0.796 KMO = 0.804^***^ Variance explained = 70.44 (%)	Product value	B1	0.547				3.36	0.815
		B2	0.642				3.28	0.987
	Functional value^*^	B3		0.838			3.36	0.833
		B4		0.847			3.41	0.860
		B5		0.875			3.28	0.907
		B6		0.859			3.16	0.888
	Service value	B7			0.602		3.44	0.740
		B8			0.831		3.75	2.387
	Price value	B9				0.881	3.86	0.782
		B10				0.859	3.79	0.896
Perceived sacrifice α = 0.924 KMO = 0.884^***^ Variance explained = 73.11 (%)	Perceived cost^*^	C1	0.680				3.16	0.890
		C2	0.932				3.40	1.022
		C3	0.942				3.40	1.016
		C4	0.941				3.33	1.005
		C5	0.907				3.31	1.000
		C6	0.677				3.42	0.988
Government participation α = 0.951 KMO = 0.759^***^ Variance explained = 91.07 (%)	Government actions	D1	0.936				4.07	0.805
		D2	0.964				4.13	0.784
		D3	0.962				4.13	0.794
Enrolment intention α = 0.949 KMO = 0.735^***^ Variance explained = 90.78 (%)	Enrolment-related actions	E1	0.968				2.93	1.138
		E2	0.969				2.82	1.098
		E3	0.921				2.99	1.072

α, Cronbach's alpha; KMO, Kaiser-Meyer-Olkin.

Measurement dimension^*^ indicates that this theoretical dimension was adjusted based on EFA.

*** p < 0.001.

(a) Involvement. To determine the measurement structure of involvement, this study was mainly based on Zaichkowsky's (23) Personal Involvement Inventory and proposed to subdivided involvement into three dimensions: product involvement, advertising involvement and enrolment-decision involvement. Specifically, product, advertising and enrolment-decision involvement respectively refer to the relevance degree to which consumers perceive the ICHI scheme itself (23), the advertising information (40) of ICHI, and the decision to enroll (41) in ICHI scheme to themselves. The items measuring each dimension were developed from the existing literature (23, 42, 43), with a total of eight items.

(b) perceived benefit and (c) perceived sacrifice. The measurement structure of perceived value has been investigated in many ways, for example, Richins and Dawson's (44) Material Values Scale (MVS) and Sweeney and Soutar's (45) Perceived Value (PERVAL) scale. In this study, we first divided perceived value into perceived benefit and perceived sacrifice. For a more systematic measurement of each construct, perceived benefit was subdivided into five dimensions: product value, service value (46), functional value, emotional value, and price value (45, 47), as well as perceived sacrifice into two dimensions: purchase cost and perceived risk (46). The items measuring each dimension were developed from the existing literature: perceived benefit (measured by ten items) (16, 45–47), perceived sacrifice (measured by six items) (19, 46).

(d) government participation (measured by three items, developed from this study); and (e) enrolment intention (measured by three items) (14, 48). The specific Likert scale items of the questionnaire are shown in Supplementary material.

### Data collection

**Figure 2:**
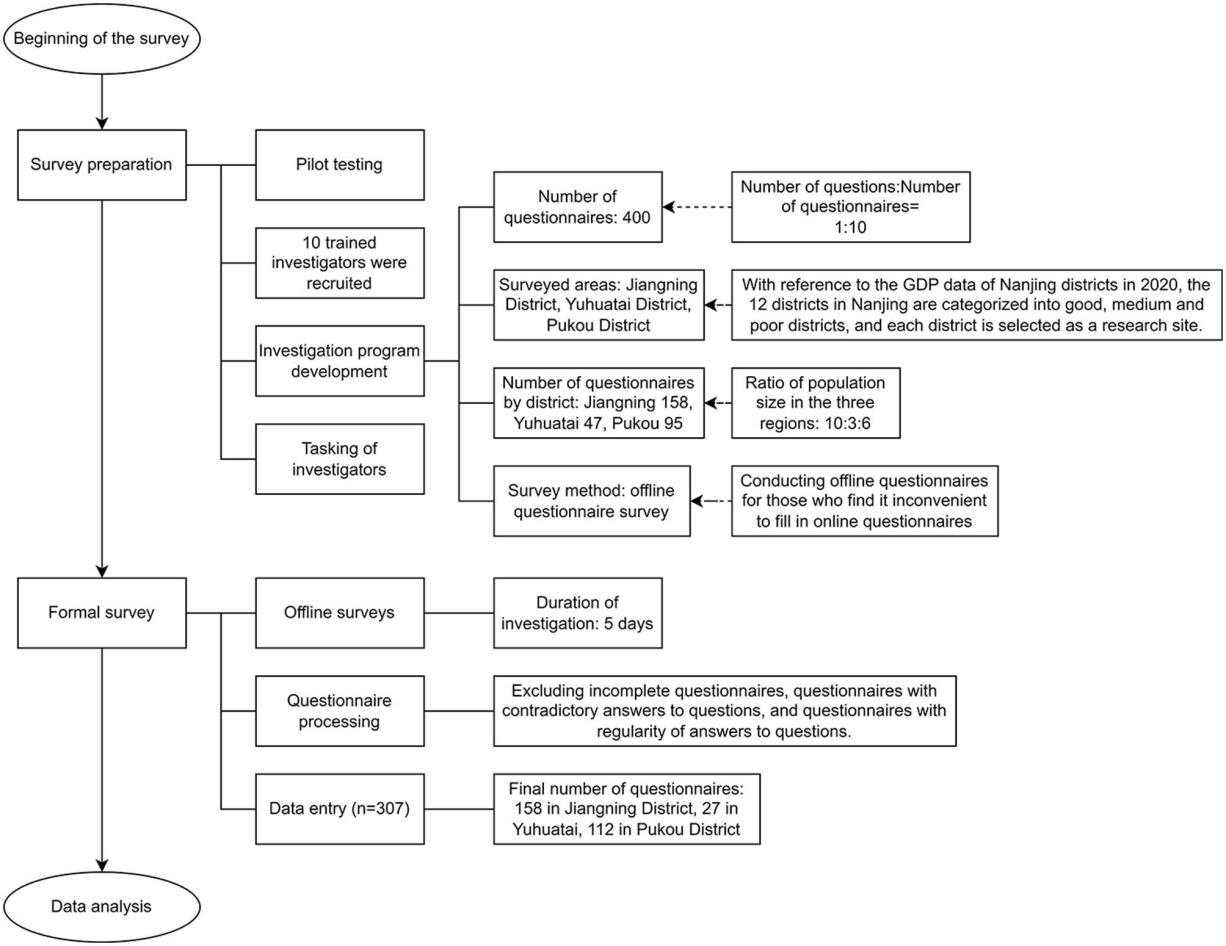
The process of data collection.

The process of data collection was shown in Figure 2. We conducted a cross-sectional survey using Nanjing as the sample area, because Nanjing is the second earliest city in China to start piloting ICHI scheme. Since the first-generation product was launched in 2018, after nearly 4 years of practice, Nanjing ICHI scheme has had a relatively higher penetration rate than other cities in China (6). As a result, the data obtained from Nanjing was believed to be more representative and meaningful for research purposes. We conducted a pilot study prior to the formal research, collecting 51 valid questionnaires with a Cronbach's alpha coefficient of 0.917 that passed the reliability test. Ten researchers received comprehensive training in research-related matters before the formal research began. To make the sample more representative of the population, a stratified random sampling method was used to divide the 12 districts of Nanjing into three tiers based on the level of economic development (with reference to the GDP of each district in 2020). One district from each tier was extracted for an offline random interview-style question-and-answer survey, and the number of questionnaires was allocated according to the number of residential population in the corresponding district. As the ICHI scheme is voluntarily purchased and based on a supplement to the basic social health insurance, all respondents are selected over 18 years of age and have basic social health insurance. The total number of distributed questionnaires was 400. Excluding incomplete surveys, surveys that mark the same box throughout the entire questionnaire, surveys with conflicting answers, surveys that have a residence outside of Jiangning, Yuhuatai, and Pukou districts in Nanjing, and surveys that lack basic medical insurance coverage in Nanjing, the total number of valid surveys collected was 307, with a validity rate of 76.75%, meeting the analysis requirements. The studies involving human participants were reviewed and approved by Ethics Committee of China Pharmaceutical University. The patients/participants provided their written informed consent to participate in this study.

### Data analysis

Prior to hypotheses testing, Exploratory Factor Analysis (EFA) using principal component analysis (PCA) with Varimax-Rotation (49) was conducted on the survey items within each construct using SPSS Version 23. This ensured the latent structure of the observed constructs and variables (50), which was necessary as these items were adopted from existing literature but were applied in the context of ICHI. Table 1 shows the factor analysis results of each research construct (the factor loadings, the mean and standard deviation (SD) of the mean for each item that was retained). The factor loadings of retained items ranged from 0.547 to 0.969. The Cronbach's Alpha of each model construct ranged from 0.796 to 0.951. The Kaiser-Meyer-Olkin (KMO) measure of adequacy for each research variable ranged from 0.735 to 0.903. The Bartlett's test of sphericity was significant for all variables. The variance explained ranged from 67.80 to 91.07%. The eigenvalues of all retained factors were all above 1. According to the results of EFA, we combined functional and emotional value into one dimension, namely functional value, and incorporated purchase cost and perceived risk into perceived cost dimension. These results showed that the items were adequate to explain the latent variables and had a good construct validity (51). Additionally, we tested for the presence of common method bias (CMB) through Harman's single factor test (52). SPSS 26 was used for conducting Exploratory Factor Analysis, where all items were included. The examination revealed that all considered measures explained 35.80% of the variance, which is significantly lower than the recommended threshold of 50% (52). Thus, CMB was not present in our study. Subsequently, descriptive statistics, one-way ANOVA, standard multiple regression, and hierarchical multiple regression were applied for analysis and hypotheses testing.

## Results

### Respondents' demographics and ANOVA

The demographics of respondents in this study are addressed in Table 2. As can be seen from the table, the sample has a gender ratio close to the true level and could be a good representation of people with a middle-level of spending power, some health problems, and some knowledge of health insurance, who are exactly the target outreach population of ICHI scheme and the main component of the risk pool. Therefore, the sample was relatively well suited to the needs of this study.

**Table 2:** Sample demographics and the analysis of enrolment intention by demographic variables using one-way ANOVA.

**Demographics**	**Proportion (%)**	**One-way ANOVA**			
		**Mean**	**SD**	***t*** **-value**	**Sig**.
**Gender**				2.013	0.157
Male	45.60	2.821	0.985		
Female	54.40	2.992	1.100		
**Age**				2.289	0.046
18–25	19.87	2.962	0.817		
26–35	34.53	3.025	1.111		
36–45	13.68	3.167	1.155		
46–55	12.70	2.821	0.964		
56–60	6.51	2.567	0.845		
>60	12.70	2.914	1.051		
**Education level**				4.710	0.001
Junior high school and below	18.57	2.626	0.928		
High school or secondary school	14.66	2.504	0.958		
College	16.29	2.973	0.992		
Bachelor's degree	42.35	3.090	1.095		
Master's degree and above	8.14	3.280	1.057		
**Whether having private health insurance**				27.328	0.000
Yes	40.72	3.277	0.968		
No	59.28	2.665	1.036		
**Disposable personal income (RMB)**				0.725	0.605
< 20,000	22.48	2.783	0.996		
20,001–40,000	12.38	2.912	0.909		
40,001–60,000	14.98	2.797	1.039		
60,001–80,000	10.75	2.879	1.133		
80,001–100,000	13.03	3.000	1.035		
>100,000	26.38	3.066	1.143		
**Self-assessment of physical health**				2.109	0.080
Very good	20.52	2.735	0.958		
Fairly good	53.75	3.004	1.063		
Medium level	22.48	2.971	1.054		
Relatively poor or very bad	3.26	2.167	1.114		
**Whether suffering from chronic disease, rare disease, and serious disease**				5.482	0.020
Yes	14.66	2.578	1.142		
No	85.34	2.972	1.026		
**Average annual personal medical expenses (RMB)**				0.300	0.878
< 5,000	80.78	2.891	1.070		
5,001–10,000	11.07	2.971	0.866		
10,001–20,000	4.23	2.949	1.008		
20,001–50,000	2.28	3.286	1.177		
>50,000	1.63	3.067	1.479		

A one-way ANOVA was conducted to examine which of the demographic variables had an impact on enrolment intention. Significant associations were found between enrolment intention and demographic variables of age, education level, whether having private health insurance, and whether suffering from chronic, rare, or serious diseases (see Table 2). Among these demographic variables, the impact of whether having private health insurance (*t* = 27.328, *p* < 0.001) was the most significant. It is also noteworthy that residents with existing private health insurance (Mean = 3.227) have a higher intention to enroll than those without other private health insurance (Mean = 2.665). Contrary to our expectations, disposable personal income was not associated with enrolment intention.

### Regression analysis

#### Test of multicollinearity

The multicollinearity test is important for multivariate analysis, which requires that the two independent variables should not perform the same work in a single regression model. The multicollinearity test method suggested by Hair et al. (53) utilized variance inflation factor (VIF) and tolerance analysis. Generally, when the tolerance is <0.20 or VIF is >5 (VIF × Tolerance = 1), it indicates that there is a multicollinearity problem (53). As shown in the Collinearity Statistics columns in Table 3, there is no multicollinearity among the independent variables of each construct in this study.

**Table 3:** Summary of coefficients for the standard multiple regression of the hypotheses testing on H1–H3.

	**Models**	**Unstandardized coefficients**		**Standardized coefficients**	***T*-test**	**Collinearity statistics**
	**Independent variables**	**B**	**Std. error**	**Beta (**β**)**	**Sig**.	**VIF**
1	Advertising involvement	0.150	0.050	0.225	0.003	3.072
	Product involvement	0.288	0.056	0.417	< 0.001	3.647
	Enrolment-decision involvement	0.071	0.044	0.087	0.012	1.664
2	Advertising involvement	−0.158	0.082	−0.186	0.055	3.072
	Product involvement	−0.168	0.092	−0.191	0.070	3.647
	Enrolment-decision involvement	0.280	0.073	0.073	< 0.001	1.664
3	Product value	0.086	0.077	0.061	0.008	1.551
	Functional value	0.499	0.075	0.373	< 0.001	1.610
	Service value	0.107	0.037	0.136	0.004	1.130
	Price value	0.326	0.072	0.248	< 0.001	1.513
4	Perceived sacrifice	−0.270	0.070	−0.216	< 0.001	1.000
5	Advertising involvement	0.039	0.074	0.036	0.003	3.072
	Product involvement	0.686	0.084	0.623	< 0.001	3.647
	Enrolment-decision involvement	0.133	0.066	0.102	0.045	1.664

The dependent variable: perceived benefit (model 1); perceived sacrifice (model 2); enrolment intention (model 3–5). Adjusted R^2^ = 0.446 for model 1; Adjusted R^2^ = 0.076 for model 2; Adjusted R^2^ = 0.399 for model 3; Adjusted R^2^ = 0.216 for model 4; Adjusted R^2^ = 0.517 for model 5.

#### Regression analysis and hypotheses testing

Table 3 presents the results of standard multiple regression tests on H1-H3. In model 1, the results demonstrated that all three dimensions of involvement: advertising involvement (β = 0.150, *p* = 0.003), product involvement (β = 0.288, *p* < 0.001), and enrolment-decision involvement (β = 0.071, *p* = 0.012) had a statistically significant positive impact on the perceived benefit (see Table 3). The largest Beta value in this model was 0.417, which is for product involvement, followed by advertising involvement (Beta = 0.225). This indicates that the product involvement variable makes the greatest unique contribution to explaining perceived benefit when the variance explained by other variables in the model was controlled. Altogether, 44.6% of the enrolment intention among residents was explained by knowing the scores for the three dimensions of Involvement (*F* = 83.173, *p* < 0.001, adjusted R^2^ = 0.446). Based on the above results, H2a was accepted.

In model 2, the regression coefficients of advertising involvement (β = −0.158, *p* = 0.055) and product involvement (β = −0.168, *p* = 0.070) in the three measurement dimensions of involvement did not pass the T-test with a significance level of 0.05. In addition, the regression model did not pass the F-test with a significance level of 0.05, and the adjusted R^2^ is only 0.076, indicating a poor model fitness. Based on the above results, it was reasonable to conclude that H2b was rejected.

In model 3, the results showed that all the four dimensions of perceived benefit: product value (β = 0.086, *p* = 0.008), functional value (β = 0.499, *p* < 0.001), service value (β = 0.107, *p* = 0.004), and price value (β = 0.326, *p* < 0.001) had a positive impact on enrolment intention. Among these dimension variables, the impact of functional value (Beta = 0.373) and price value (Beta = 0.248) was relatively stronger. Altogether, the four dimensions of perceived benefit can explain 39.9% of the change in enrolment intention (*F* = 51.884, *p* < 0.001, adjusted R^2^ = 0.399). Therefore, H1a was accepted.

Model 4 indicated that perceived sacrifice (β = −0.207, *p* < 0.001) had a negative impact on the enrolment intention (*F* = 14.889, *p* < 0.001, adjusted R^2^ = 0.216), and results in model 5 showed that all the three dimensions of involvement: advertising involvement (β = 0.039, *p* = 0.003), product involvement (β = 0.686, *p* < 0.001), and enrolment-decision involvement (β = 0.133, *p* = 0.045) had a significant positive impact on enrolment intention. In addition, the F-test results and adjusted R^2^ in model 5 (*F* = 110.039, *p* < 0.001, adjusted R^2^ = 0.517) indicated a good model fitness. Based on the above results, H1b and H3 were accepted. Furthermore, it was discovered that product involvement showed a stronger influence capability in both model 1 (Beta = 0.417) and model 5 (Beta = 0.623).

### Mediation effect analysis

As per Baron and Kenny's (54) approach, mediating effect is tested in the following steps (X: independent variable; Y: dependent variable; M: mediator variable): (1) regress M on X, and look for a significant coefficient associated with X; (2) regress Y on X, and look for a significant coefficient associated with X; (3) regress Y on X and M, and look for a significant coefficient associated with M, then the mediating effect was demonstrated when the effect of X on Y was less in the third regression equation than in the second.

For H4b, it has been found that involvement (independent variable) does not affect perceived sacrifice (mediator variable) in the upper subsection. As a result, H4b was rejected. Then, for the test of H4a, model 6–7 were used for the steps 1–2 respectively, and they all had good fitness and significant regression coefficients [model 6: β for X (involvement) = 0.528, *p* < 0.001, adjusted R^2^ = 0.444; model 7: β for X (involvement) = 0.883, *p* < 0.001, adjusted R^2^ = 0.489] (see Table 4). Then model 8 shows the regression results in the test of step (3). The mediator variable perceived benefit had a significant effect on enrolment intention (β = 0.437, *p* < 0.001), and the effect of involvement on enrolment intention was less than the effect in the regression model 7, as evidenced by a decrease in the regression coefficient (β_model 8_ = 0.652 < β_model 7_ = 0.883). Thus, all the assumptions of Baron and Kenny's method were satisfied, and H4a was accepted.

**Table 4:** Summary of coefficients in the standard multiple regression for mediating effect analysis.

	**Models**	**Dependent variable**	**Model coefficients**			**Model summary**
	**Independent variables**		β	**Std. Error**	**Sig**.	**Adjusted R** ^2^
6	Involvement	Perceived benefit	0.528	0.034	0.000	0.444^***^
7	Involvement	Enrolment intention	0.883	0.052	0.000	0.489^***^
8	Involvement	Enrolment intention	0.652	0.066	0.000	0.529^***^
	Perceived benefit		0.437	0.084	0.000	

*** p < 0.001.

### Moderating effect analysis

According to the model test method proposed by Wen-Zhonglin and Hou (55) involving both mediator variables and moderator variables, this study adopts the sequential test method to verify the moderating effect of the moderated mediation model involved in this study, which is divided into four steps (X: independent variable; Y: dependent variable; M: mediator variable; W: moderator variable): (1) regress Y on X and W, and look for a significant coefficient associated with X; (2) regress M on X and W, and look for a significant coefficient associated with X; (3) regress Y on X, W, and M; and look for a significant coefficient associated with M; (4) regress Y on X, W, M, and W×M; A significant coefficients associated with W×M suggests that the relationship between M and Y is moderated by W.

Table 5 shows the results of the regression analysis. Model 9–11 were used for the tests of steps 1–3 respectively, and they all had good fitness and significant regression coefficients [model 9: β for X (involvement) = 0.832, *p* < 0.001, adjusted R^2^ = 0.521; model 10: β for X (involvement) = 0.492, *p* < 0.001, adjusted R^2^ = 0.484; model 11: β for M (perceived benefit) = 0.356, *p* < 0.001, adjusted R^2^ = 0.545]. After including an interaction term for perceived benefit and government participation, model 10 still showed significant regression results and good model fitness [β for W×M (Government participation × Perceived benefit) = 0.209, *p* = 0.02 < 0.05, adjusted R^2^ = 0.576]. Thus, all the assumptions of Wen-Zhonglin and Hou's (55) method were satisfied, implying that government participation positively moderates the effect of perceived benefit on enrolment intention. Therefore, H5a was accepted.

**Table 5:** Summary of coefficients in the standard multiple regression for moderating effect analysis.

	**Models**	**Dependent variables**	**Model coefficients**			**Model summary**
	**Independent variables**		β	**Std. error**	**Sig**.	**Adjusted R** ^2^
9	Involvement	Enrolment intention	0.832	0.051	0.000	0.521^***^
	Government participation		0.260	0.056	0.000	
10	Involvement	Perceived benefit	0.492	0.033	0.000	0.484^***^
	Government participation		0.181	0.037	0.000	
11	Involvement	Enrolment intention	0.657	0.065	0.000	0.545^***^
	Government participation		0.195	0.057	0.001	
	Perceived benefit		0.356	0.086	0.000	
12	Involvement	Enrolment intention	0.646	0.065	0.000	0.576^***^
	Government participation		−0.507	0.305	0.097	
	Perceived benefit		−0.517	0.382	0.177	
	Government participation × Perceived benefit		0.209	0.089	0.020	
13	Perceived sacrifice	Enrolment intention	−0.278	0.066	0.000	0.552^***^
	Government participation		0.462	0.073	0.000	
14	Perceived sacrifice	Enrolment intention	−0.272	0.060	0.027	0.557^***^
	Government participation		0.460	0.070	0.002	
	Perceived sacrifice × Government participation		−0.154	0.005	0.005	

*** p < 0.001.

Having determined that perceived sacrifice is not a mediator variable between involvement and enrolment intention, hierarchical regression analysis was conducted to examine the moderating effect of government participation on perceived sacrifice and enrolment intention. Model 13–14 in Table 5 illustrates the results of hierarchical regression analysis. After adding the moderator variable Government participation to the regression model, model 13 showed that government participation had a positive influence on enrolment intention (β = 0.462, *p* < 0.001). When including an interaction term for perceived sacrifice and government participation (model 14), regression coefficient of perceived sacrifice × government participation was significant (β = −0.154, *p* = 0.005). The results indicated that government participation had a negative moderating effect of perceived sacrifice on enrolment intention. H5b was thus accepted.

### Modification of the theoretical model

**Figure 3:**
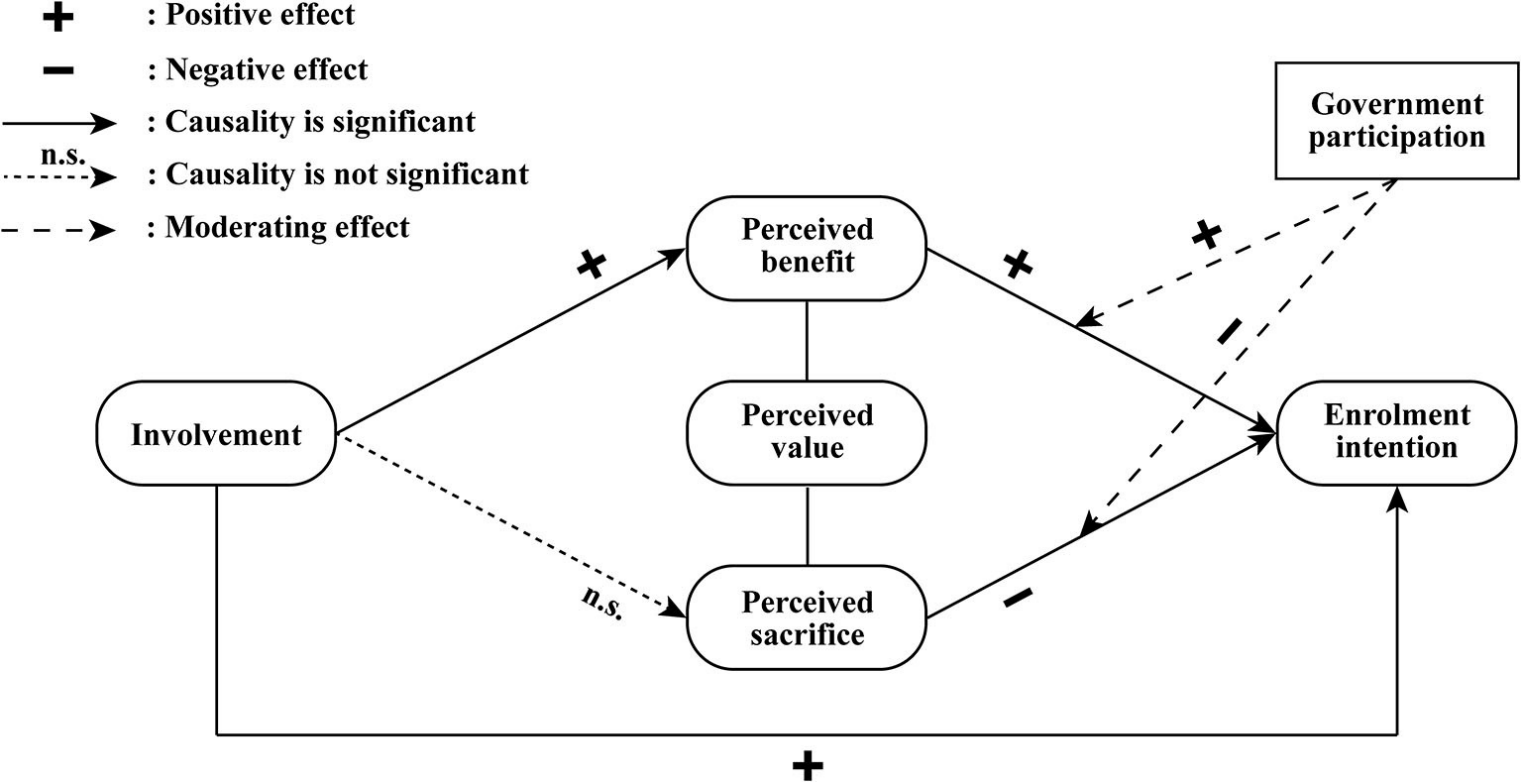
Conceptual model of the factors influencing residents' enrolment intention.

To sum up, results of the analysis above showed that the hypotheses proposed in this study are valid except for H2b and H4b. Figure 3 shows the revised conceptual model.

## Discussion

The findings from this study provide a more holistic picture of the multidimensional factors that influence residents' enrolment intention as well as their influencing mechanisms, from a demand-side perspective. In exploring the influence of control variables, we found that there are demographic variables (e.g. age, education level, health status, and existing level of insurance coverage) that influenced residents' enrolment intention, and the impact of existing level of insurance coverage was the most significant. Among the demographic variables studied in this study, it is noteworthy that income level has no effect on enrolment intention. This could be explained by the low premiums of ICHI scheme, which account for a relatively small percentage of residents' total income. To conclude, in the future promotion to more cities, designers of ICHI scheme should pay increased attention to the influence of the differing individual characteristics of residents in different cities on their intentions to enroll. Moreover, our findings also provided additional evidence and pilot directions for many domestic scholars who advocate the city-differentiated customized insurance schemes (6, 12).

Then, based on the revised conceptual model of the psychographic factors influencing enrolment intention, we found the following: First, we discovered a statistically significant positive impact of involvement on both perceived benefit and enrolment intention. Reflecting on the difference in the influence capability of three dimensions of involvement, it is noted that the impact of product involvement is the strongest. This indicated that more attention should be paid to the product design to make it more related to residents' lives in future development, because this factor is not only an instrumental pre-factor for the formation of residents' value perception, but also a significant driving factor for increased enrolment intention. These findings strengthened the conclusions of existing studies (30, 33, 34) and further complemented the differing influence capabilities of the different dimensions of involvement as well.

Second, our research revealed that involvement does not affect residents' perceived sacrifice, and the mediating effect of perceived sacrifice between involvement and enrolment intention is therefore not significant. This might seem to be in conflict with Venkatraman's research (29). On the other hand, residents' perceived benefit was found to mediate the effect of involvement on enrolment intention. These findings suggested that the reason why residents' enrolment intention increases with the deepening of involvement is that they are more aware of benefits brought by ICHI scheme, rather than their decreased concern over potential sacrifices. One possible explanation is that the overall involvement among residents is currently low. According to the study of Maheswaran and Meyers-Levy (26), individuals in the low involvement condition often draw and apply the inference that they agree more with issues associated with positive rather than negative information, while under high involvement individuals would presumably assign more weight to the negatively framed rather than the positively framed information and be more persuaded by it. This explanation was also consistent with the descriptive statistical analysis of each model construct variable that the average score of involvement was the lowest (see Table 1). Thus, at the current stage, policymakers and product providers should seek to improve the overall involvement of residents, and to achieve this, increasing product involvement by optimizing product design to make it more relevant to residents' lives is considered the most beneficial approach based on the influence capability analysis above.

Third, our results indicated that perceived benefit positively affects residents' enrolment intention while perceived sacrifice negatively affects their enrolment intention, which was in line with the basic connotation of the perceived value theory. In addition, empirical analysis based on further subdivisions of different research dimensions showed that functional value and price value have a more significant impact on enrolment intention. In contrast, the impact of service value and product value is relatively weak. For one thing, product designers can further improve residents' perceived benefit by optimizing existing functional value and price value, as well as expanding additional functional value such as value-added services. For another, product promoters can employ better publicity strategies to reduce residents' enrolment concerns.

Lastly, we found that government participation positively moderates the effect of perceived benefit on enrolment intention. It is conceivable that under the national conditions of China, residents have a trust relationship with the government. This means that involving the government can strengthen residents' perception of the benefits of ICHI scheme, thereby increasing their intention to enroll. However, contrary to our expectation, government participation negatively moderates the effect of perceived sacrifice on enrolment intention. Considering that there have been few quantitative studies on government participation in ICHI scheme, additional research is required to determine the causes of this negative moderating effect. Our future study will refine the measurement dimensions of the government participation variable and take into account factors such as different intervention channels and publicity methods to examine the moderating effect of government participation in greater depth. Last but not least, since government participation does have a significant moderating effect, it is recommended for policymakers to clarify the functional positioning of each participating government department.

On the one hand, this paper further complemented the differing capabilities of the different dimensions of involvement and perceived benefit to influence enrolment intentions, expanding the research boundaries of involvement theory and perceived value theory. On the other hand, research targeting the enhanced sustainability of health insurance programs will provide a practical model for other social health insurance-led countries to link social insurance and private insurance, with the aim of alleviating the growing financial pressure of social health insurance as a single payer. However, the current study is not without limitations. First, this study was limited in scope by several factors, including the epidemic situation, and we only selected one of the most representative cities, Nanjing, to serve as the survey sample area in this case. Consequently, the research conclusions may have certain regional characteristics instead of being universal. Second, in the absence of well-established scales for measuring government participation variables, the questionnaire development in this section lacks a solid theoretical and practical basis. This may lead to empirical data that may not accurately reflect government participation actions. Lastly, the involvement theory and the perceived value theory are primarily used in the construction of the conceptual model. This may result in that not all influencing factors and intermediate variables are included, such as residents' attitudes toward the product. Future studies could incorporate the attitude variables from the Theory of Planned Behavior, and comprehensively consider the formation mechanism of residents' enrolment intention.

## Conclusion

Although ICHI scheme has been rapidly promoted in China under the joint efforts of the government and insurance companies, the overall enrolment rate of residents remains low currently. While existing studies have mostly focused on the supply-side perspective, this study proposed that it is equally important to understand the multidimensional factors that influence residents' behavioral intention from a demand-side perspective. By examining these factors and their interrelationships, this study developed a multivariate conceptual model of the psychographic process by which residents' enrolment intentions are formed. Based on this model, we suggested ways for health policymakers and promoters to increase the enrolment among residents. Through analysis, this study has identified that involvement and the two constructs of perceived value (i.e. perceived benefit and perceived sacrifice) are all crucial psychographic process factors that influence the formation of residents' enrolment intentions, and that government participation is a moderator of this psychographic process. When analyzing the interrelationships between the five constructs in the model, two findings deserve special attention: First, perceived benefit mediates the effect of involvement on enrolment intention, while perceived sacrifice does not. This result indicated that one of the main dilemmas in the current development of ICHI scheme is the low level of involvement among residents. Second, government participation positively moderates the influence path of “perceived benefit—enrolment intention” but negatively moderates the path of “perceived sacrifice—enrolment intention”. Further research is required to understand the opposite moderating direction of government participation as a moderator. At the current stage, policymakers and product providers should seek to improve the overall involvement of residents, and to achieve this, increasing product involvement by optimizing product design to make it more relevant to residents' lives is considered the most beneficial approach.

## Data availability statement

The original contributions presented in the study are included in the article/Supplementary material, further inquiries can be directed to the corresponding authors.

## Ethics statement

Written informed consent was obtained from the individual(s) for the publication of any potentially identifiable images or data included in this article.

## Author contributions

TS, YW, JX, RS, and RJ contributed to the study design. TS, YW, and XH made contributions to the acquisition, analysis, and interpretation of data. TS prepared the first draft of the manuscript. YW, RS, and RJ made substantial revisions to the manuscript prior to its submission. All authors have approved the submitted version.
