# Supplementary material for: Correlates of residents' enrolment intention toward inclusive commercial health insurance in China: involvement, perceived benefit, perceived sacrifice, and government participation
Source: Front Public Health. 2023 Oct 30;11:1121783. doi: 10.3389/fpubh.2023.1121783 (PMC10643177; doi:10.3389/fpubh.2023.1121783)
Supplement: Supplementary file 1 [file Table_1.DOCX]

Supplementary Material

**Supplementary Table 1.** Constructs, measurement dimensions and survey items modified by Exploratory Factor Analysis.

| **Constructs** | **Measurement**  **Dimensions** | **Codes** | **Survey Items** |
| --- | --- | --- | --- |
| **Involvement** | Advertising  involvement | A1 | The promotional message of Inclusive Commercial Health Insurance scheme is attractive to me. |
|  |  | A2 | The promotional message of Inclusive Commercial Health Insurance scheme is meaningful and valuable to me. |
|  |  | A3 | The promotional message of Inclusive Commercial Health Insurance scheme will make me take the initiative to learn more about this insurance product. |
|  | Product  involvement | A4 | Inclusive Commercial Health Insurance scheme is attractive to me. |
|  |  | A5 | Inclusive Commercial Health Insurance scheme is meaningful and valuable to me. |
|  |  | A6 | I need Inclusive Commercial Health Insurance scheme |
|  | Enrolment-decision  involvement | A7 | It is necessary to spend time and effort to understand and enrol in Inclusive Commercial Health Insurance scheme. |
|  |  | A8 | Collecting information or asking others about enrolling in Inclusive Commercial Health Insurance scheme can help me make a enrolment decision. |
| **Perceived**  **benefit** | Product value | B1 | The guarantee clauses of Inclusive Commercial Health Insurance scheme are easy to understand. |
|  |  | B2 | The claim settlement process of Inclusive Commercial Health Insurance scheme is convenient. |
|  | Functional value | B3 | Being insured with Inclusive Commercial Health Insurance scheme makes my life more secure. |
|  |  | B4 | Being insured with Inclusive Commercial Health Insurance scheme can reduce the possibility of poverty due to illness. |
|  |  | B5 | Being insured with Inclusive Commercial Health Insurance scheme makes me feel at ease. |
|  |  | B6 | Being insured with Inclusive Commercial Health Insurance scheme makes me feel happy. |
|  | Service value | B7 | The service of enrolling in Inclusive Commercial Health Insurance scheme is good. |
|  |  | B8 | Insurance companies offering Inclusive Commercial Health Insurance scheme are trustworthy. |
|  | Price value | B9 | The pricing of Inclusive Commercial Health Insurance scheme is reasonable. |
|  |  | B10 | Inclusive Commercial Health Insurance scheme is cost-effective. |
| **Perceived**  **sacrifice** | Perceived cost | C1 | I am worried that collecting and understanding the relevant information of Inclusive Commercial Health Insurance scheme will consume a lot of my time and energy. |
|  |  | C2 | I am worried that Inclusive Commercial Health Insurance scheme has a cumbersome and complicated claim settlement process. |
|  |  | C3 | I am worried that the waiting period for completing the claim settlement of Inclusive Commercial Health Insurance scheme may be very long. |
|  |  | C4 | I am worried that Inclusive Commercial Health Insurance scheme cannot fulfil its guaranteed responsibilities entirely. |
|  |  | C5 | I am worried that the subsequent claim amount of Inclusive Commercial Health Insurance scheme will not meet my psychological expectations. |
|  |  | C6 | I am worried that the Inclusive Commercial Health Insurance scheme I have taken out does not fully meet my needs. |
| **Government**  **participation** | Government actions | D1 | The work the government has done to enhance the promotion of Inclusive Commercial Health Insurance scheme is important. |
|  |  | D2 | The work the government has done to strengthen the supervision during the operation of Inclusive Commercial Health Insurance scheme is important. |
|  |  | D3 | The work the government has done to participate in the process of product design of Inclusive Commercial Health Insurance scheme is important. |
| **Enrolment intention** | Enrolment-related actions | E1 | I am willing to take out Inclusive Commercial Health Insurance scheme. |
|  |  | E2 | I am willing to take out Inclusive Commercial Health Insurance scheme on an ongoing basis. |
|  |  | E3 | I am willing to recommend others to take out Inclusive Commercial Health Insurance scheme. |
